# Dissimilatory Sulfate Reduction Under High Pressure by *Desulfovibrio alaskensis* G20

**DOI:** 10.3389/fmicb.2018.01465

**Published:** 2018-07-09

**Authors:** Adam J. Williamson, Hans K. Carlson, Jennifer V. Kuehl, Leah L. Huang, Anthony T. Iavarone, Adam Deutschbauer, John D. Coates

**Affiliations:** ^1^Department of Plant and Microbial Biology, University of California, Berkeley, Berkeley, CA, United States; ^2^Energy Biosciences Institute, Berkeley, CA, United States; ^3^Environmental Genomics and Systems Biology, Lawrence Berkeley National Laboratory, Berkeley, CA, United States; ^4^QB3/Chemistry Mass Spectrometry Facility, University of California, Berkeley, Berkeley, CA, United States

**Keywords:** sulfate reduction, souring, sulfidogenesis, oil reservoirs, perchlorate, nitrate

## Abstract

Biosouring results from production of H_2_S by sulfate-reducing microorganisms (SRMs) in oil reservoirs. H_2_S is toxic, corrosive, and explosive, and as such, represents a significant threat to personnel, production facilities, and transportation pipelines. Since typical oil reservoir pressures can range from 10 to 50 MPa, understanding the role that pressure plays in SRM metabolism is important to improving souring containment strategies. To explore the impact of pressure, we grew an oil-field SRM isolate, *Desulfovibrio alaskensis* G20, under a range of pressures (0.1–14 MPa) at 30°C. The observed microbial growth rate was an inverse function of pressure with an associated slight reduction in sulfate and lactate consumption rate. Competitive fitness experiments with randomly bar-coded transposon mutant library sequencing (RB-TnSeq) identified several genes associated with flagellar biosynthesis and assembly that were important at high pressure. The fitness impact of specific genes was confirmed using individual transposon mutants. Confocal microscopy revealed that enhanced cell aggregation occurs at later stages of growth under pressure. We also assessed the effect of pressure on SRM inhibitor potency. Dose-response experiments showed a twofold decrease in the sensitivity of *D. alaskensis* to the antibiotic chloramphenicol at 14 MPa. Fortuitously, pressure had no significant influence on the inhibitory potency of the common souring controlling agent nitrate, or the emerging SRM inhibitors perchlorate, monofluorophosphate, or zinc pyrithione. Our findings improve the conceptual model of microbial sulfate reduction in high-pressure environments and the influence of pressure on souring inhibitor efficacy.

## Importance

Sulfate-reducing microorganisms (SRMs) are ubiquitous in many deep subsurface environments that are exposed to high pressure and are particularly problematic in oil reservoirs due to the production of sulfide, which is toxic and corrosive. Therefore, understanding SRM activity under high pressure is important to understand global sulfur cycling in the deep subsurface and may help to improve existing souring inhibitor treatment strategies. Here, we describe competitive fitness experiments that identify several flagellar genes important for fitness and show evidence for enhanced biofilm formation under high pressure. Importantly, pressure does not significantly impact the efficacy of several known SRM inhibitors, however, we observed enhanced resistance to the antibiotic chloramphenicol, which may have implications for small molecule inhibitor resistance and should be further investigated. Our findings improve our understanding of sulfate reduction and the impact of stress on this important group of microorganisms.

## Introduction

The production of H_2_S by SRM *in situ* or in the produced fluids of an oil reservoir (denoted souring) often occurs as a result of seawater injection during secondary oil recovery operations (Gieg et al., 2011). Due to the toxic, explosive, and corrosive nature of H_2_S, souring poses significant health, facility, and environmental damage risks (Gieg et al., 2011) with a total estimated annual cost exceeding $90 billion. Nitrate inhibits sulfate reduction in laboratory studies (Callbeck et al., 2013; Carlson et al., 2014) and some oil fields, but its success in the field is unpredictable and often unsuccessful (Gieg et al., 2011). Alternative inhibitors such as perchlorate and monofluorophosphate (MFP) are potent SRM inhibitors in the lab (Carlson et al., 2014, 2015; Engelbrektson et al., 2014; Gregoire et al., 2014; Mehta-Kolte et al., 2017), however, for successful field implementation, souring treatment strategies based on these emerging technologies require a comprehensive understanding of SRM metabolism over a range of environmental conditions.

Oil exploration and reservoir development continue to occur in progressively deeper formations and enhanced oil recovery efforts are increasingly undertaken over a wide range of depths. To enhance success of souring control, both current and future oil recovery efforts will require an accurate understanding of pressure-dependence of SRM metabolism. However, relatively few studies have assessed the influence of pressure effects on SRM (Pradel et al., 2013; Amrani et al., 2014; Wilkins et al., 2014) and no study has evaluated the efficacy of souring treatment strategies at high-pressure. Sedimentary basins have been explored to depths up to 7 km below the surface and many discoveries have occurred at 1–4 km (Planckaert, 2005). With average pressure gradients of 1–2.5 MPa and temperature gradients of 3°C per 100 m, the temperature and pressure in these deeper reserves may surpass life-limiting extremes, at 200°C and 100 MPa, respectively. However, the majority of discovered deep oil reservoirs are more moderate in their temperature and pressure profiles with ranges from 50 to 150°C and 10–50 MPa. Even at the shallowest of these deeper reservoirs (1 km), microbial processes may be significantly influenced by the environmental conditions (50°C and 10 MPa) (Planckaert, 2005).

Pressure is a physical and thermodynamic parameter that can affect gas solubility and redox potentials (Takai et al., 2008; Picard et al., 2012), and thereby influence chemical equilibria and reaction rates. High pressure can inhibit cellular processes such as cell division (Welch et al., 1993) and can change osmotic potential gradients to increase cell permeability and alter protein hydration to enhance protein stability and change enzyme activity (Bartlett, 2002; Huang et al., 2016). Flagellar motility is often inhibited by high pressures (Meganathan and Marquis, 1973; Eloe et al., 2008; Nishiyama et al., 2013), but the reasons for this are largely unknown.

Three SRM with optimal growth pressures in the range of 10–40 MPa have been isolated, namely *Desulfovibrio profundus*, *D. piezophilus*, and *D. hydrothermalis* (Bale et al., 1997; Alazard et al., 2003; Khelaifia et al., 2011). Pressure resistance mechanisms of these piezophilic organisms include alterations of membrane fluidity by fatty acid packing modifications, osmolyte accumulation to stabilize charge imbalance and macromolecule conformation, and modifications in energy metabolism (Pradel et al., 2013; Amrani et al., 2014). High pressure is not tolerated by all SRM, however, and has been shown to be inhibitory to strains of *D. salexigens* and *D. alaskensis* (formerly *desulfuricans*) between 5 and 20 MPa (Bale et al., 1997). No further characterization of this pressure-induced stress was reported and to our knowledge, no studies to date have assessed gene fitness and survival mechanisms of piezosensitive SRM under high pressure. In the context of an oil reservoir undergoing secondary oil recovery, piezosensitive SRM are likely introduced into the reservoir environment as a result of seawater injection, and thus, understanding the genes that govern piezoresistance of these organisms to high pressure is of great interest. One study has focused on the impact of CO_2_ on *D. vulgaris* Hildenborough under high pressure (Wilkins et al., 2014). No impact on cell growth under N_2_ was observed up to 8 MPa, however, in CO_2_-flushed media, growth was significantly inhibited above 2.5 MPa and supercritical CO_2_ in these systems fragmented lipids that compromised membrane integrity, and triggered EPS formation as a defense mechanism (Wilkins et al., 2014).

Herein, we report new insights into high-pressure fitness mechanisms of a model SRM, *D. alaskensis* G20, originally isolated from an oil well corrosion site in Ventura County, California. Also, we evaluated the impact of pressure on the efficacy of a range of SRM inhibitors. While we did not see a change in the efficacy of most souring treatments, we did identify genes involved in flagellar motility and cell aggregation as important for resistance of *D. alaskensis* to high pressure. Thus, preventing biofilm formation is likely an additional strategy to deal with SRM piezosensitivity in high-pressure soured oil systems.

## Results and Discussion

### Impact of High Pressure on Growth and Sulfate Reduction of *D. alaskensis* G20

**FIGURE 1:**
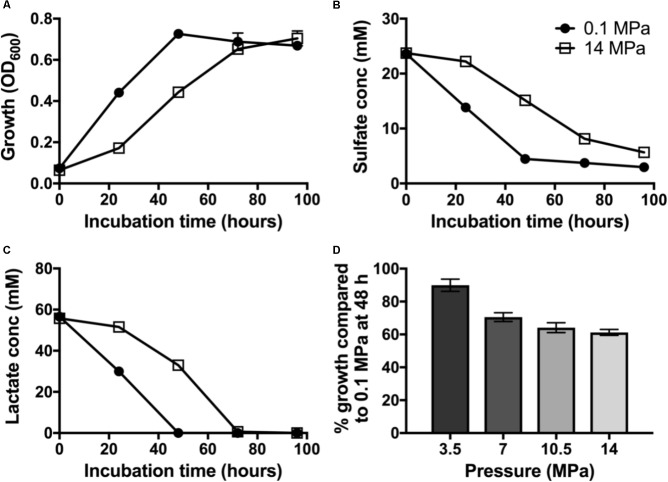
Dissimilatory sulfate reduction at 0.1 (•) and 14 MPa (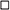) of *Desulfovibrio alaskensis* G20: **(A)** planktonic growth, **(B)** sulfate consumption, **(C)** lactate consumption, and **(D)** % growth of G20 after 48 h incubation at 3.5, 7, 10.5, and 14 MPa compared to atmospheric pressure control.

To evaluate the impact of pressure on piezo-responsive SRM physiology, we compared the growth and sulfate reduction rates of wild-type *D. alaskensis* G20 at low and high pressures. At 0.1 MPa, a maximum OD of 0.72 ± 0.03 was achieved at 48 h (**Figure 1A**) and an average growth rate (μ) of 0.062 ± 0.003 h^−1^. We observed complete lactate consumption (59.03 ± 1.03 mM) by 48 h and maximum sulfate consumption (25.1 ± 2.4 mM) by the final time point (**Figures 1B,C**). At 14 MPa, no statistical difference in maximum OD at 96 h (0.70 ± 0.04) was observed, but a ∼25% slower growth rate (0.046 ± 0.007 h^−1^) was noted. At high pressure, complete lactate consumption (59.8 ± 1.4 mM) by 72 h, and sulfate reduction (23.3 ± 0.9 mM) occurred by 96 h of incubation.

To investigate the pressure threshold at which the growth of *D. alaskensis* G20 is impacted, we measured growth via cell density (OD_600_) at 48 h in cultures grown at increasing discrete pressures (3.5, 7, 10.5, and 14 MPa). We observed a stepwise decrease in the growth at 48 h in cultures grown at pressures above 7 MPa relative to 3.5 MPa (**Figure 1D**). This result is consistent with previous studies using this organism and the closely related *D. salexigens* (Bale et al., 1997).

### Identifying Genes Important for High Pressure Tolerance and Sensitivity in *D. alaskensis* G20

**FIGURE 2:**
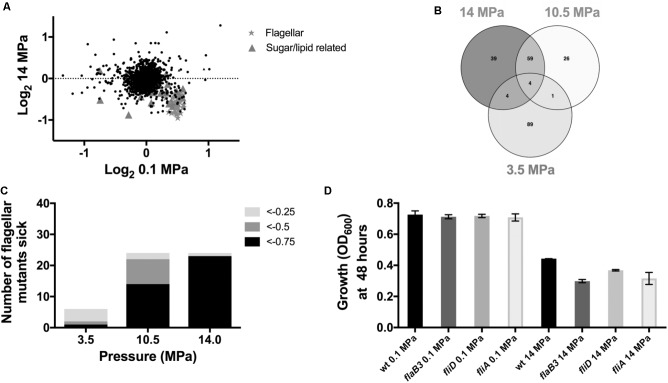
Mutant fitness under high pressure: **(A)** Correlation plot of 0.1 MPa vs. 14 MPa from the competitive fitness experiments. **(B)** Venn diagram to show fitness defects >–0.5 shared between 3.5, 10.5, and 14 MPa. **(C)** Number of flagellar mutants sick with increasing pressure and **(D)** transposon mutant growth experiments.

To identify genes whose disruption perturbs growth at high pressure relative to ambient pressure, we grew transposon mutant library pools of *D. alaskensis* G20 at 0.1, 3.5, 10.5, and 14 MPa. These transposon mutants contain DNA barcodes that enable the estimation of gene fitness for all non-essential genes in parallel using a competitive growth assay, as previously described (Kuehl et al., 2014). Transposon mutants which have insertions in specific genes with a fitness value (log_2_ ratio of the strain abundance after growth compared to the initial inoculation) of less than −0.5 in both the 10.5 and 14 MPa experiments versus the low (3.5 MPa) or 0.1 MPa controls were considered important for fitness at high pressure. Comparison of the gene fitness scores for 0.1 and 14 MPa cultures, which showed the greatest difference in growth rate, identified several genes involved in flagellar biosynthesis as important for growth at high pressure (**Figure 2A** and **Supplementary Table S1**). Additionally, two Fe-S cluster proteins (Dde_3200 and 3201) and a multidrug resistance protein (mrp; Dde_3202), that has homology with MinD and ParA involved in cell division, were also important for fitness at 14 and 10.5 MPa (**Supplementary Table S2**). Noteworthy mutations in genes resulting in beneficial pressure fitness included two glutamate synthase genes (Dde_1218 and 3635) (**Supplementary Figure S1** and **Supplementary Table S4**). Proteomics data indicated that glutamine and glutamate synthase proteins gltA (Dde_3635) and glnA (Dde_0104) were also downregulated under pressure (**Supplementary Table S3**). Glutamate has been implicated as an osmolyte to stabilize intracellular macromolecules against high pressure induced alterations to, e.g., protein conformation, packing and interactions (Martin et al., 2002; Amrani et al., 2014) and salt stress (Mukhopadhyay et al., 2006). The accumulation in cells is generally associated with downregulation of glutamate synthase proteins, which apparently are more often involved in glutamate catabolism rather than synthesis (Amrani et al., 2014). Our observations are consistent with these findings.

In further support of a role for flagellar motility in response to high pressure, 59 genes were important at both 10.5 and 14 MPa (**Figure 2B** and **Supplementary Table S2**) and over a third of these shared genes were associated with flagellar biosynthesis and assembly. In general, genes annotated as involved in flagellar biosynthesis were increasingly important for survival at escalating pressures. At 14, 10.5, and 3.5 MPa, 96, 58, and 17%, respectively, of flagellar genes had fitness scores less than −0.75 (**Figure 2C**). No flagellar genes were important for survival at 0.1 MPa. To confirm the importance of flagellar for survival of G20 at high pressure, we tested several individual transposon mutant strains (*Δfla*B3, *Δfli*D, and *Δfli*A) for growth at 14 MPa. No significant difference in growth was observed in any of the mutants tested at atmospheric pressure (**Figure 2D**) and all mutants were non-motile as confirmed by microscopy. At 14 MPa pressure, however, all flagellar mutants grew to a lower OD_600_ by 48 h compared to the parental G20 strain (**Figure 2D**). Furthermore, all flagellar mutants tested had a lower average growth rate and a lower maximum final OD_600_ than the atmospheric pressure control (**Supplementary Table S5**).

Flagellar motility is a critical function for nutrient acquisition, biofilm formation, and movement away from stress (O’Toole et al., 2000; McCarter, 2006). Flagellar biosynthesis pathways have been implicated for a number of environmental stresses including salt (He et al., 2010), pH (Stolyar et al., 2007), and temperature (Wall et al., 2007) in a close relative to *D. alaskensis* G20: *D. vulgaris* Hildenborough. Flagellar biosynthesis has also been associated with chemotaxis, highlighting their importance in oxygen, nitric oxide, nitrate, and carbon dioxide avoidance (Fu and Voordouw, 1997; Wall et al., 2007; Pereira et al., 2008; Wilkins et al., 2014). Links between flagellar biosynthesis and biofilm formation have also been reported during syntrophic with *D. alaskensis* G20 (Krumholz et al., 2015).

Flagellar regulation is also often observed as a pressure-sensitive cellular process, but the regulatory mechanism and importance for fitness either varies or is not well characterized in most organisms. An increase in motility at high pressure by the piezophile *Photobacterium profundum* has been linked to the enhanced flagellar rotation (Eloe et al., 2008). The upregulation of flagella under high pressure in transcriptomes in deep ocean environments is suggested to be due to a greater need for surface attachment in deeper waters (Delong et al., 2006; Eloe et al., 2008). In contrast, *E. coli* motility is inhibited at high pressure, but the mechanism is not well understood (Nishiyama and Sowa, 2012).

**FIGURE 3:**
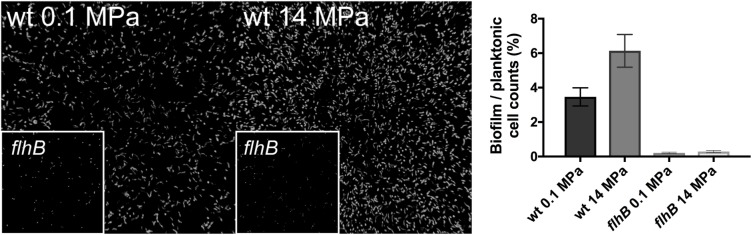
Confocal microscope images of wild type G20 at 0.1 MPa **(Left)** and 14 MPa **(Center)** after 48 h of incubation. *flhB* transposon mutants at 0.1 and 14 MPa are shown as an inset of each panel. Measured cell counts of cells affixed to glass slides (biofilm) versus calculated planktonic cells from OD_600_ (planktonic) for wild type and *flhB* at 0.1 and 14 MPa are shown in the **Right**.

Motility is important for biofilm formation in a number of microorganisms (O’Toole et al., 2000; McCarter, 2006), including SRM in the *Desulfovibrio* genus (Clark et al., 2007) and growth in a biofilm could plausibly confer high pressure resistance. Thus, we hypothesized that G20 biofilm formation would be important to resist higher pressures and thus enhanced survival. To determine the importance of flagella for biofilm formation at high pressure, we incubated glass slides in cultures with and without applied pressure. Qualitatively, there was significantly more biomass associated with the glass slide surfaces at 14 MPa relative to 0.1 MPa after 48 and 72 h of incubation (**Figure 3**). In support of this observation, cell count ratios of cells affixed to glass slides versus planktonic cells was almost twofold higher under pressure (6.1 ± 0.9%) compared to the atmospheric control (3.5 ± 0.5) (**Figure 3**). Transposon mutant cells of the flagellar biosynthetic protein *flh*B were non-motile and no cell aggregation was observed at any pressure tested (**Figure 3**). This result is consistent with previous studies using flagella-deficient mutants (Clark et al., 2007). Very few studies have established the link between high pressure and induction of biofilm formation, but cell aggregation was observed in *P. phosphoreum* at high pressure (∼22 MPa) (Martini et al., 2013), and an uncharacterized “slime” was observed in a high-pressure corrosion study with a sulfate-reducing isolate (Herbert and Stott, 1983). Our results strongly suggest a link between motility, biofilm formation, and high-pressure fitness in sulfate-reducing bacteria.

Biofilm formation is a complex multi-step process dependent on flagellar motility, extracellular polymeric substances (EPS) production, and cellular metabolic state (O’Toole et al., 2000; McCarter, 2006). To identify these additional factors, we searched for other possible markers of biofilm formation in our gene fitness datasets. We found two genes (Dde_3587 and 3588) encoding for OmpA domain proteins that were also important for G20 survival at both 14 and 10.5 MPa (**Supplementary Table S2**). These membrane porins associated with the flagellar motor stator MotB, are in a predicted operon with several flagellar related genes and have been implicated in *E. coli* biofilm formation on hydrophobic surfaces (Ma and Wood, 2009). Several other genes possibly related to EPS production were also important for growth at both 14 and 10.5 MPa, but not 3.5 MPa. These included three genes of the heptosyltransferase family (Dde_0565, Dde_1714, and Dde_2022), several glycosyltransferases (Dde_0337 and Dde_0426), a glycerol-3-phosphate dehydrogenase (Dde_3178), and a UDP gene (Dde_2187) (**Supplementary Table S1**). Extracellular polymeric substances (EPS) can provide mechanical stability to biofilms, as well as facilitating water and nutrient retention that can be beneficial for bacteria attachment to surfaces (Sutherland, 2001). High pressure has also been shown to stimulate EPS and biofilm formation in *Colwellia psychrerythraea*, suggesting a role for EPS as a survival strategy (Marx et al., 2009). Extensive EPS formation has also been reported as a protective mechanism in response to CO_2_ exposure at high pressure (Wilkins et al., 2014). To determine if genes important for biofilm formation were upregulated in response to high-pressure, we compared proteomes from 0.1 to 14 MPa cultures and identified pressure-dependent upregulation of another OmpA domain protein (Dde_1689) and a glycerol-3-phosphate dehydrogenase protein (gap; Dde_3736) (**Supplementary Table S3**). We attempted to quantify and compare EPS production in our cultures, but did not observe significant differences in levels of soluble sugars between 0.1 and 14 MPa cultures at experimental endpoints. We cannot rule out the role of EPS formation, however, as EPS is typically associated with the biofilm matrix, thus further work is needed to quantify EPS within the biofilm fraction.

### Evaluating the Influence of High Pressure on SRM Inhibitor Efficacy

Antibiotic resistance by biofilms has been widely documented (Høiby et al., 2010) due to their ability to act as a physical barrier that may slow antibiotic penetration, or deactivation of antibiotics in surface layers by EPS chelation of organic and inorganic ions and redox-active metals (Flemming and Wingender, 2010). As such, it is reasonable to hypothesize that the enhanced biofilm forming capacity observed under pressure for *D. alaskensis* could lead to greater antibiotic resistance at higher pressure. In support of this, dose-response experiments with chloramphenicol revealed that the IC_50_ at 14 MPa increased to 22 μM (19–27 μM), compared to atmospheric pressure of 9 μM (7.1–13.6 μM) (**Supplementary Figure S2**). Furthermore, no pressure dependency in inhibition was observed with the non-biofilm forming *flh*B transposon mutant [IC_50_ values of 3.6 μM (2.4–5.5 μM) and 5.9 μM (3.5–10.0 μM) for 0.1 and 14 MPa, respectively] (**Supplementary Figure S2**), suggesting that the G20 resistance to chloramphenicol at high pressure is a function of biofilm formation. While chloramphenicol is an antibiotic which primarily targets the ribosome, it is known to promote cell aggregation in *Salmonella* (Brunelle et al., 2015). It is possible that a similar response could occur in *Desulfovibrio* endowing it with a decreased sensitivity to chloramphenicol.

Similar experiments were performed to evaluate the impact of pressure on the sensitivity of *D. alaskensis* G20 to existing (nitrate) and an emerging selective inhibitor (perchlorate) commonly used in the oil and gas industry. Nitrate is commonly used in oil reservoirs as an effective biocompetitive inhibitor of sulfate reduction (Hubert and Voordouw, 2007; Callbeck et al., 2013). Nitrate respiration also results in the production of nitrite which is a very potent inhibitor of the dissimilatory sulfite reductase, a prerequisite enzyme to SRM (Hubert et al., 2005). Perchlorate is emerging as an attractive, more effective, potent, and predictable alternative to nitrate. Perchlorate also functions via biocompetitive exclusion in the presence of perchlorate reducing microorganisms. However, both perchlorate and nitrate at high concentrations (>10 mM) are also direct competitive inhibitors of the ATP-sulfurylase, another highly conserved prerequisite enzyme in sulfate reduction pathway (Carlson et al., 2014). Similarly, although not biocompetitive electron acceptors, we also tested two direct inhibitors of SRM (MFP and zinc pyrithione). MFP is a highly effective competitive inhibitor of the ATP-sulfurylase (Carlson et al., 2015), but also releases cytoplasmic fluoride as a result of intracellular hydrolysis enhancing its toxic effect (Carlson et al., 2015). Zinc pyrithione is a potent biocide that displays some selectivity for sulfate reducers via glutathione or Cu transport, (Carlson et al., 2017), but the mode of operation still awaits further determination. The IC_50_ values reported for each of these inhibitors tested at 0.1 MPa were consistent with previous aforementioned studies (**Table 1** and **Supplementary Figure S2**). In contrast to observations made with chloramphenicol, there was no significant (greater than twofold change of mean IC_50_) pressure dependency on their respective potencies at 14 MPa (**Table 1** and **Supplementary Figure S2**), suggesting that their effect is likely unimpeded in high pressure environments.

**Table 1:** Concentrations of diverse inhibitors that inhibit 50% of *D. alaskensis* G20 activity at 0.1 and 14 MPa.

Compound	IC_50_ 0.1 MPa (mM)	IC_50_ 14 MPa (mM)
Nitrate	58 (48–71)	56 (50–63)
Perchlorate	30 (28–32)	21 (18–24)
MFP	2.2 (1.9–2.4)	2.8 (2.7–2.9)
Zinc pyrithione	0.021 (0.017–0.025)	0.023 (0.019–0.026)
Chloramphenicol	0.009 (0.007–0.014)	0.022 (0.019–0.027)

*Values in parenthesis are confidence intervals.*

## Conclusion and Significance

We show how individual gene fitness for a sulfate-reducing bacterium, *D. alaskensis* G20 is impacted by pressure. We highlight the response of this microorganism to pressure and show that flagellar genes are important for fitness under pressure and have confirmed this phenotype using individual transposon mutants. Additionally, EPS genes were important for survival at high pressure. Enhanced cell aggregation was observed using confocal microscopy and these observations establish a link between pressure, flagella, and biofilm formation. Biofilm formation is particularly problematic in oil production facilities as protein filaments can attach onto the anodic sites of metals and enhance microbial-influenced corrosion (MIC) (Wikieł et al., 2014; Kip and van Veen, 2015). The impact of pressure on biofilm formation and MIC clearly warrants further work. Furthermore, our observations of enhanced resistance of *D. alaskensis* G20 to chloramphenicol at high pressure further corroborates the link between pressure, flagella, and biofilm formation. Although antibiotics are not used to treat sulfidogenesis, our fundamental insights of biofilm resistance may have implications toward other small molecule SRM inhibitors and may warrant further work. Importantly, we saw no influence of high pressure on inhibition by nitrate, perchlorate, MFP, or zinc pyrithione, compounds with existing and potential applicability in souring control. Future work should investigate the impact of other antibiotics and biocides under pressure. This study presents important data on the genes important for high pressure resistance in piezo-responsive SRM, and establishes important causal links between high pressure response, motility, biofilm formation, and inhibitor resistance. Our results will improve our conceptual models of piezo-responsive SRM physiology in high-pressure environments such as soured oil reservoirs or the deep ocean.

## Materials and Methods

### Growth of *Desulfovibrio alaskensis* G20 and Biogeochemical Analyses

*Desulfovibrio alaskensis* strain G20 was cultivated in anoxic basal Tris-buffered lactate/sulfate media, pH 7.4 at 30°C, as previously described (Carlson et al., 2015). Prior to any experiment performed in this study, wild type and selected transposon mutants of *D. alaskensis* G20 were recovered from 1 ml freezer stocks in 10 ml anoxic basal media with 1 g L^−1^ yeast extract and 1 mM sodium sulfide. *D. alaskensis* G20 was then subsequently transferred into fresh lactate/sulfate media, before transferring into sterile plastic Luer Lok syringes and sealed with a polyvinyl cap. All growth experiments were conducted in triplicate. High pressure experiments were conducted in a stainless steel pressure vessel fitted with two thermostat controlled heat mats. Samples were loaded into the vessel with a BD GasPak anaerobic catalyst and the vessel was flushed with high purity (99.9%) N_2_ for 15 min. Vessel pressure was then increased at a rate of ∼0.5 MPa min^−1^ using high purity N_2_. Atmospheric pressure control samples were stored in a BD GasPak anaerobic box with a BD GasPak catalyst (BD, Franklin Lakes, NJ, United States). Growth (OD_600_) could not be measured *in situ* under pressure however. Instead, at experimental timepoints, the pressure vessel was depressurized (∼0.5 MPa min^−1^) and syringes were shaken and 1 ml samples were removed from syringes for measurements of planktonic growth at OD_600_, sulfate using ion chromatography (Dionex ICS 1500, Dionex, Sunnyvale, CA, United States) and lactate using HPLC (Dionex LC20, Dionex, Sunnyvale, CA, United States). The specific growth rate (μ h^−1^) was calculated from the change in OD_600_ between exponential phase time points.

### Pooled Transposon Assays and Individual Transposon Mutant Experiments

1 ml frozen (−80°C) aliquots of tagged-transposon pools of *D. alaskensis* G20 (Kuehl et al., 2014) were recovered in MOLS media with 1 g L^−1^ yeast extract and 1 mM sodium sulfide, centrifuged and washed to remove residual yeast extract and resuspended in an initial OD_600_ of 0.02 in 10 ml fresh media containing 1 mM sodium sulfide. When pools reached an OD_600_ ∼0.8 (5–6 doublings), 2 ml aliquots were collected via centrifugation and stored at −20°C until genomic DNA extraction. Genomic DNA was extracted with a Qiagen (Redwood city, CA, United States) DNAeasy kit following the procedure for extraction of genomic DNA from Gram-negative bacteria. The optional RNAse treatment step was included. DNA barcodes were then PCR amplified and sequenced on an Illumina machine as previously described (Wetmore et al., 2015). Gene fitness was calculated as previously described as the log_2_ ratio of the strain abundance after growth compared to the initial inoculation, averaged over duplicate samples (Kuehl et al., 2014). Reported fitness difference values are log_2_ (applied pressure −0.1 MPa control). Genes with fitness difference >1 were considered to have beneficial mutations and those with fitness difference <−1 were considered to have detrimental mutations. Individual transposon mutant strains were recovered from frozen stocks in an archived strain collection (Kuehl et al., 2014) and grown on anaerobic MoYLS4 media + 400 μg ml^−1^ G418 antibiotic and incubated for 3 days at 30°C in an anaerobic chamber. Individual colonies were then picked into 1 ml LS + 800 μg ml^−1^ G418 antibiotic and grown for 2 days. All mutants were confirmed for identity by PCR before growth experiments were conducted.

### Proteomics

Cells for proteomics were harvested from triplicate 50 mL mid-log phase cultures (OD_600_ 0.3–0.5). Cells were pelleted anaerobically at 4000 rcf, supernatant was decanted and cells were resuspended and digested according to the following procedure. 50 μL of a 1 μg/μL lysate sample was placed in a capped low-retention microcentrifuge tube. Ten microliters of 100 mM NH_4_HCO_3_, pH 7.5 was added along with 25 μL of a 0.2% Rapigest SF solution. The sample was placed in a block heater set to 80°C and heated for 15 min. The sample was removed from the block heater, centrifuged and 10 μL of 100 mM dithiothreitol was added and the samples were heated to 60°C for 30 min. Ten microliters of 100 mM iodoacetamide was added and the samples kept in the dark for 30 min at room temperature. Samples were digested overnight at 37°C by addition of 10 μL of 0.5 μg/μL Trypsin Gold porcine protease (Promega). The following morning, 10 μL of 0.5% trifluoroacetic acid was added to quench digests and samples were incubated at 37°C for 30 min. Precipitated cell debris was pelleted by centrifugation at 14,000 RPM, 6°C for 30 min and the supernatant containing soluble peptides was transferred into a new low-retention microcentrifuge tube. Samples were filtered through 0.2 μm cellulose syringe filters (National Scientific F2404-16) and kept in autosampler vials at 4°C until analysis by liquid chromatography-tandem mass spectrometry (LC-MS/MS) methods described in Carlson et al. (2014). Briefly, Trypsin-digested proteins were analyzed using a Thermo Dionex UltiMate3000 RSLCnano LC that was connected in-line with an LTQ Orbitrap XL mass spectrometer equipped with a nanoelectrospray ionization (nanoESI) source (Thermo Fisher Scientific, Waltham, MA, United States). A linear gradient elution was conducted with 99.9% water/0.1% formic acid (solvent A) and 99.9% acetonitrile/0.1% formic acid (v/v) (solvent B).

Data acquisition was controlled using Xcalibur software (version 2.0.7 SP1, Thermo). Raw data files were searched against the *D. alaskensis* strain G20 protein database using Proteome Discoverer software (version 1.3, SEQUEST algorithm, Thermo) for tryptic peptides with up to three missed cleavages, carboxyamidomethylcysteine as a static post-translational modification, and methionine sulfoxide as a variable post-translational modification. A decoy database was used to characterize the false discovery rate and the target false discovery rate was 0.01 (i.e., 1%). Peptide counts were normalized by dividing the number of peptides observed for a given protein in a sample by the total number of peptides observed in that sample. Normalized peptide counts were log_2_ transformed and raw reads and processing are reported in SI. We identified a total of 775 proteins in these experiments. *t*-Tests are reported for all differences in protein counts between 0.1 and 14 MPa, with *p*-values < 0.05 representing a significant difference.

### Biofilm Imaging

Biofilm formation by *D. alaskensis* was assessed using laser scanning confocal microscopy (Carl Zeiss Inc., LSM710). Sterile glass microscope slides were submerged into the culture media prior to incubation. At experimental end-points, glass slides were removed, washed gently with phosphate buffered saline (PBS) and then fixed in 4% formaldehyde in PBS. Slides were then stained with SYTO BC Green Fluorescent Nucleic acid stain (Thermo Fisher). Biofilm cell counts were conducted using the bubbles function of IMARIS 7.0 software (Bitplane AG).

### Dose-Response Experiments

*Desulfovibrio alaskensis* G20 was inoculated in 2× concentrated MOLS media (OD_600_ 0.04), then subsequently mixed in a 1:1 ratio with a series of twofold dilutions of inhibitors [perchlorate, nitrate, MFP, zinc pyrithione, and chloramphenicol (Sigma Aldrich, St Louis, MO, United States)] in 96-deep well micro-plates to give a final OD_600_ of 0.02 in 1× MOLS media. The culture and inhibitor were subsequently transferred to 1 ml Luer Lok syringes, capped and incubated at 0.1 and 14 MPa. Experiments were sampled after 48 h for cell turbidity at OD_600._ Data analysis for inhibition experiments was carried out in GraphPad Prism 6 (GraphPad Software Inc., La Jolla, CA, United States) and curves were fit to a standard inhibition log dose-response curve to generate IC_50_ values. Ninety five percent confidence intervals are reported. IC_50_ values are reported as the mean of three biological replicates.

## Author Contributions

JC conceived and directed the project, and co-authored the manuscript. AW performed the research, interpreted the data, and wrote the manuscript. HC aided in the research and data interpretation. JK, LH, AI, and AD aided in the research and provided the analytical tools.

## Conflict of Interest Statement

The authors declare that the research was conducted in the absence of any commercial or financial relationships that could be construed as a potential conflict of interest.

## Supplementary Material

FIGURE S1 Venn diagram to show fitness >+0.5 shared between 3.5, 10, and 14 MPa.

FIGURE S2 IC_50_ plots for ClO_4_^−^, NO_3_^−^, Zinc pyrithione, MFP, chloramphenicol and IC_50_ values of chloramphenicol for wild type and *flhB* strains of G20.

TABLE S1 Flagellar genes sick at 14 MPa.

TABLE S2 Venn analysis of data from competitive fitness experiments to show detrimental mutations shared between all pressures tested.

TABLE S3 Proteomic data to show proteins up and downregulated in wild-type *D. alaskensis* G20 at 14 MPa.

TABLE S4 Venn analysis of data from competitive fitness experiments to show beneficial mutations shared between all pressures tested.

TABLE S5 Average growth rate and maximum OD_600_ of *Desulfovibrio alaskensis* G20 wild type (wt), and transposon mutants *flaB3*, *fliD* and *fliA* at 0.1 MPa and 14 MPa.
