# Supplementary figures and images for: Dissimilatory Sulfate Reduction Under High Pressure by Desulfovibrio alaskensis G20
Source: Front Microbiol. 2018 Jul 9;9:1465. doi: 10.3389/fmicb.2018.01465 (PMC6052904; doi:10.3389/fmicb.2018.01465)

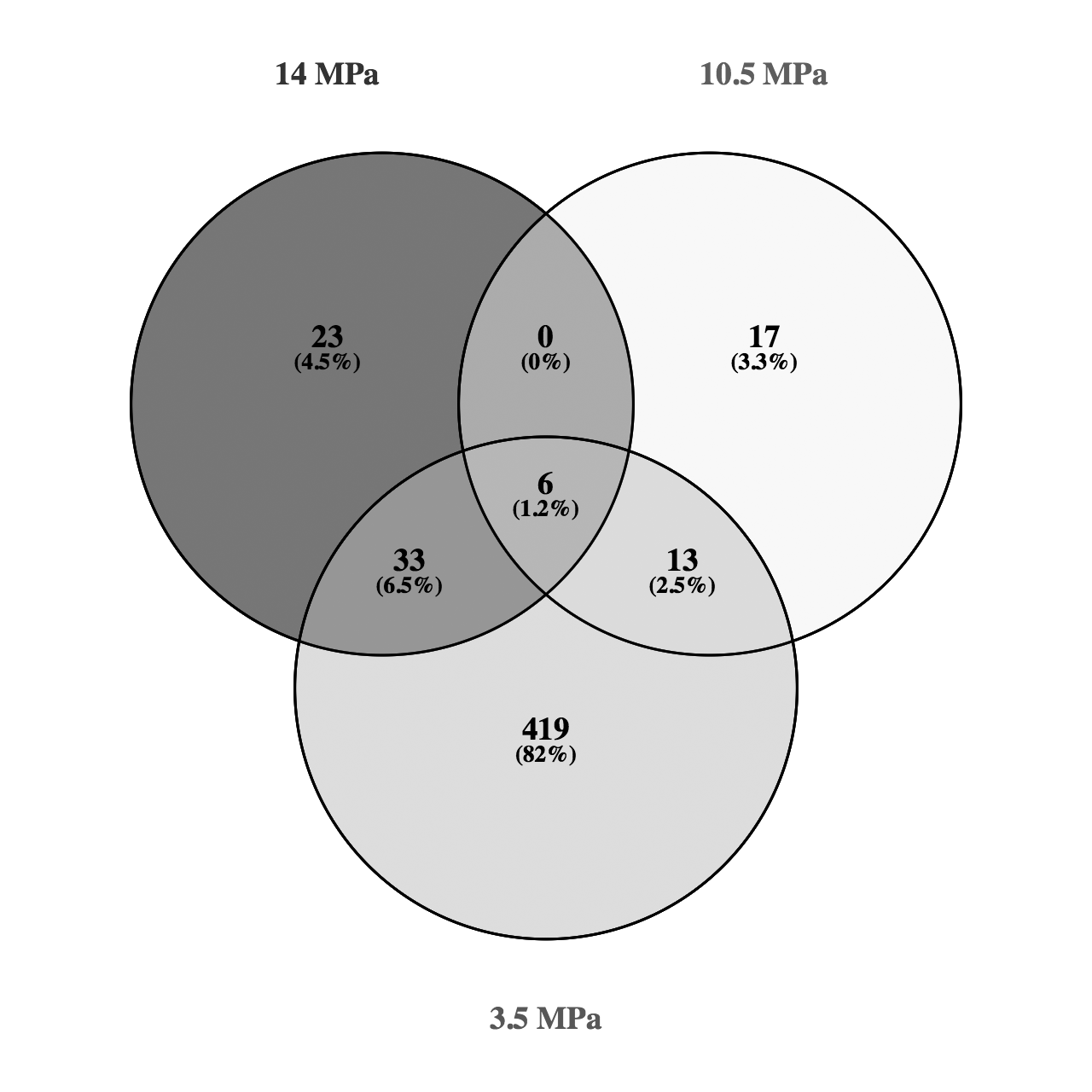

Supplement: FIGURE S1 — Venn diagram to show fitness >+0.5 shared between 3.5, 10, and 14 MPa. [file Image_1.TIF]

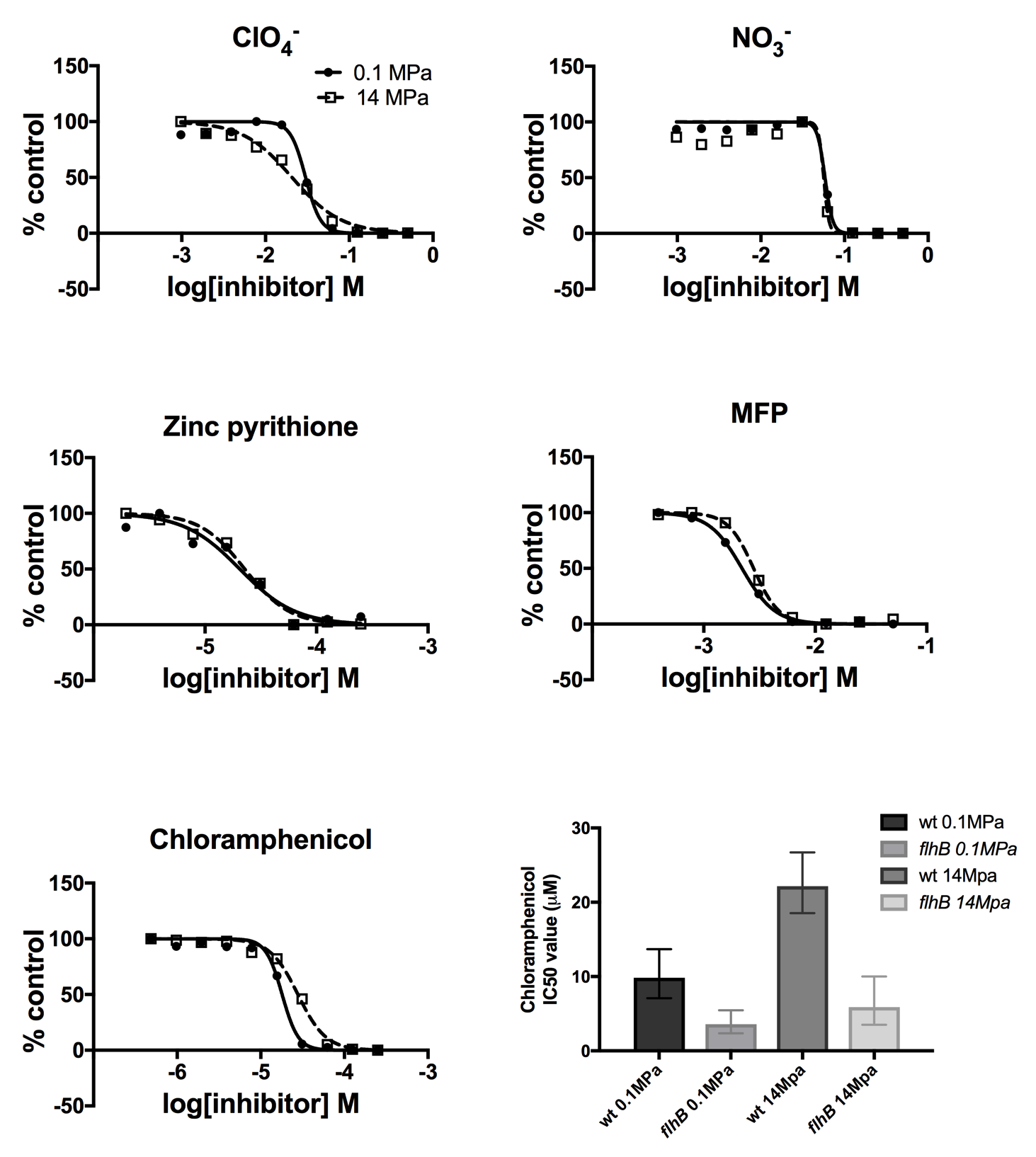

Supplement: FIGURE S2 — IC50 plots for ClO4−, NO3−, Zinc pyrithione, MFP, chloramphenicol and IC50 values of chloramphenicol for wild type and flhB strains of G20. [file Image_2.TIFF]
