# Supplementary material for: Dissimilatory Sulfate Reduction Under High Pressure by Desulfovibrio alaskensis G20
Source: Front Microbiol. 2018 Jul 9;9:1465. doi: 10.3389/fmicb.2018.01465 (PMC6052904; doi:10.3389/fmicb.2018.01465)
Supplement: TABLE S1 — Flagellar genes sick at 14 MPa. [file Table_1.docx]

**Supporting Information Table 1: Flagellar genes sick at 14 MPa**

| Gene | 0.1 Mpa | 14 Mpa | Fitness difference 14 Mpa-0.1 Mpa |
| --- | --- | --- | --- |
| Dde_1120 395567 Flagellar biosynthesis; filament capping protein; enables filament assembly, FliD (VIMSS-AUTO) | 0.51 | -0.95 | -1.46 |
| Dde_0298 393133 Flagellar biosynthesis, hook protein (VIMSS-AUTO) | 0.55 | -0.84 | -1.39 |
| Dde_0357 3333984 Flp pilus assembly protein TadD | 0.49 | -0.87 | -1.37 |
| Dde_0355 393082 Flagellar basal-body rod protein FlgC | 0.48 | -0.86 | -1.33 |
| Dde_0350 393087 Flagellum-specific ATP synthase (VIMSS-AUTO) | 0.59 | -0.71 | -1.30 |
| Dde_0352 393085 Flagellar motor switch protein FliG | 0.60 | -0.69 | -1.29 |
| Dde_0356 393081 Flagellar basal body protein FlgB | 0.53 | -0.76 | -1.29 |
| Dde_3157 393867 Flagellar basal body P-ring biosynthesis protein | 0.47 | -0.81 | -1.29 |
| Dde_3156 393868 Flagellar biosynthesis, basal-body outer-membrane L (lipopolysaccharide layer) ring protein (VIMSS-AUTO) | 0.45 | -0.82 | -1.26 |
| Dde_0351 393086 Flagellar assembly protein FliH domain protein (VIMSS-AUTO) | 0.60 | -0.63 | -1.23 |
| Dde_0379 393060 Flagellar biosynthetic protein FlhB (VIMSS-AUTO) | 0.48 | -0.73 | -1.22 |
| Dde_0380 393059 Flagellar biosynthesis protein FlhA | 0.51 | -0.69 | -1.21 |
| Dde_3152 393872 Flagellar hook-associated protein FlgM, putative (VIMSS-AUTO) | 0.48 | -0.70 | -1.19 |
| Dde_1502 395254 Flagellin (flaB3) (VIMSS-AUTO) | 0.36 | -0.81 | -1.17 |
| Dde_3151 393873 Flagellar hook-associated protein 3, putative (VIMSS-AUTO) | 0.47 | -0.70 | -1.17 |
| Dde_3159 393865 Flagellar basal-body rod protein FlgF (flgF) (VIMSS-AUTO) | 0.48 | -0.66 | -1.14 |
| Dde_3583 393537 Flagellar biosynthetic protein FliP | 0.54 | -0.58 | -1.11 |
| Dde_0353 393084 flagellar M-ring protein FliF | 0.55 | -0.52 | -1.07 |
| Dde_0383 3333988 Flagellar biosynthesis protein FliA | 0.52 | -0.55 | -1.07 |
| Dde_0378 393061 Flagellar biosynthetic protein FliR | 0.52 | -0.54 | -1.06 |
| Dde_1119 395568 Flagellar biosynthetis protein FliS, putative (VIMSS-AUTO) | 0.47 | -0.57 | -1.04 |
| Dde_3582 393538 Flagellar biosynthetic protein FliQ | 0.59 | -0.40 | -0.99 |
| Dde_1712 395064 Flagellar biosynthesis, initiation of hook assembly (VIMSS-AUTO) | 0.38 | -0.58 | -0.96 |
| Dde_3585 393535 Flagellar motor switch protein FliN (fliN) (VIMSS-AUTO) | 0.50 | -0.43 | -0.93 |
| Dde_2959 394039 Flagellar regulatory protein A (flrA) (VIMSS-AUTO) | 0.46 | -0.45 | -0.91 |
| Dde_1709 395067 Flagellin (flaB1) (VIMSS-AUTO) | 0.42 | -0.47 | -0.89 |
| Dde_3586 393534 Flagellar protein FliL, putative (VIMSS-AUTO) | 0.55 | -0.32 | -0.87 |
| Dde_3158 393866 Flagellar biosynthesis, cell-distal portion of basal-body rod (VIMSS-AUTO) | 0.46 | -0.36 | -0.82 |
| Dde_1711 395065 Flagellar hook protein FlgE | 0.31 | -0.47 | -0.78 |
| Dde_0381 393058 Flagellar biosynthesis protein FlhF, putative | 0.13 | -0.31 | -0.43 |
