# Supplementary material for: Dissimilatory Sulfate Reduction Under High Pressure by Desulfovibrio alaskensis G20
Source: Front Microbiol. 2018 Jul 9;9:1465. doi: 10.3389/fmicb.2018.01465 (PMC6052904; doi:10.3389/fmicb.2018.01465)
Supplement: TABLE S2 — Venn analysis of data from competitive fitness experiments to show detrimental mutations shared between all pressures tested. [file Table_2.DOCX]

**Supporting Information Table 2: Venn analysis of data from competitive fitness experiments to show detrimental mutations shared between all pressures tested**

| **Genes shared between 14 Mpa and 10.5 Mpa** | **fitness difference 14 Mpa** | **fitness difference 10.5 Mpa** |  |
| --- | --- | --- | --- |
| Dde_0298 393133 Flagellar biosynthesis, hook protein (VIMSS-AUTO) | -1.39 | -0.67 |  |
| Dde_0357 3333984 Flp pilus assembly protein TadD | -1.37 | -0.57 |  |
| Dde_1713 395063 Conserved hypothetical protein (VIMSS-AUTO) | -1.35 | -0.76 |  |
| Dde_0355 393082 flagellar basal-body rod protein FlgC | -1.33 | -0.96 |  |
| Dde_0350 393087 Flagellum-specific ATP synthase (VIMSS-AUTO) | -1.30 | -0.75 |  |
| Dde_0352 393085 flagellar motor switch protein FliG | -1.29 | -0.77 |  |
| Dde_0356 393081 Flagellar basal body protein FlgB | -1.29 | -0.80 |  |
| Dde_3157 393867 Flagellar basal body P-ring biosynthesis protein | -1.29 | -0.84 |  |
| Dde_3156 393868 Flagellar biosynthesis, basal-body outer-membrane L (lipopolysaccharide layer) ring protein (VIMSS-AUTO) | -1.26 | -0.80 |  |
| Dde_0564 392896 Conserved hypothetical protein (VIMSS-AUTO) | -1.24 | -0.75 |  |
| Dde_0351 393086 Flagellar assembly protein FliH domain protein (VIMSS-AUTO) | -1.23 | -0.73 |  |
| Dde_0379 393060 Flagellar biosynthetic protein FlhB (VIMSS-AUTO) | -1.22 | -0.74 |  |
| Dde_0380 393059 flagellar biosynthesis protein FlhA | -1.21 | -0.80 |  |
| Dde_3152 393872 Flagellar hook-associated protein FlgM, putative (VIMSS-AUTO) | -1.19 | -0.67 |  |
| Dde_3201 393827 Iron-sulfur cluster binding protein (VIMSS-AUTO) | -1.19 | -1.22 |  |
| Dde_1502 395254 Flagellin (flaB3) (VIMSS-AUTO) | -1.17 | -0.71 |  |
| Dde_3151 393873 Flagellar hook-associated protein 3, putative (VIMSS-AUTO) | -1.17 | -0.76 |  |
| Dde_3159 393865 Flagellar basal-body rod protein FlgF (flgF) (VIMSS-AUTO) | -1.14 | -0.96 |  |
| Dde_3583 393537 Flagellar biosynthetic protein FliP | -1.11 | -1.18 |  |
| Dde_0565 392895 Heptosyltransferase family (VIMSS-AUTO) | -1.11 | -0.73 |  |
| Dde_0216 393205 H04M03.4 gene product (VIMSS-AUTO) | -1.11 | -0.88 |  |
| Dde_2022 394803 Heptosyltransferase family (VIMSS-AUTO) | -1.09 | -0.85 |  |
| Dde_1486 395270 Glycosyl transferase , group 2 family protein domain | -1.09 | -0.76 |  |
| Dde_0353 393084 flagellar M-ring protein FliF | -1.07 | -1.01 |  |
| Dde_0337 393098 Glycosyl transferase, group 2 family protein domain protein (VIMSS-AUTO) | -1.07 | -0.62 |  |
| Dde_0378 393061 flagellar biosynthetic protein FliR | -1.06 | -0.91 |  |
| Dde_2930 394064 dTDP-4-dehydrorhamnose 3,5-epimerase | -1.05 | -0.90 |  |
| Dde_1119 395568 Flagellar biosynthetis protein FliS, putative (VIMSS-AUTO) | -1.04 | -0.66 |  |
| Dde_1571 395190 Protein-glutamate methylesterase CheB (cheB-2) (VIMSS-AUTO) | -1.04 | -1.35 |  |
| Dde_2351 394505 Conserved hypothetical protein | -1.03 | -0.74 |  |
| Dde_2706 394208 Conserved hypothetical protein | -1.02 | -1.00 |  |
| Dde_3155 393869 Homolog of Salmonella P-ring of flagella basal body (VIMSS-AUTO) | -1.02 | -0.99 |  |
| Dde_3582 393538 flagellar biosynthetic protein FliQ | -0.99 | -1.05 |  |
| Dde_3584 393536 Conserved hypothetical protein (VIMSS-AUTO) | -0.98 | -1.36 |  |
| Dde_1714 395062 Heptosyltransferase family (VIMSS-AUTO) | -0.98 | -0.69 |  |
| Dde_1712 395064 Flagellar biosynthesis, initiation of hook assembly (VIMSS-AUTO) | -0.96 | -0.60 |  |
| Dde_3587 393533 OmpA family domain protein (VIMSS-AUTO) | -0.95 | -0.87 |  |
| Dde_2187 394648 UDP-glucose 4-epimerase (VIMSS-AUTO) | -0.95 | -0.76 |  |
| Dde_3585 393535 Flagellar motor switch protein FliN (fliN) (VIMSS-AUTO) | -0.93 | -1.41 |  |
| Dde_2960 394038 TPR Domain domain protein (VIMSS-AUTO) | -0.92 | -0.85 |  |
| Dde_2959 394039 Flagellar regulatory protein A (flrA) (VIMSS-AUTO) | -0.91 | -1.02 |  |
| Dde_3154 393870 Membrane proteins related to metalloendopeptidases (VIMSS-AUTO) | -0.89 | -0.68 |  |
| Dde_3586 393534 Flagellar protein FliL, putative (VIMSS-AUTO) | -0.87 | -1.32 |  |
| Dde_3588 393532 OmpA family domain protein (VIMSS-AUTO) | -0.84 | -0.90 |  |
| Dde_2573 394315 Conserved hypothetical protein (VIMSS-AUTO) | -0.83 | -0.70 |  |
| Dde_0439 393006 Putative glycosyl/glycerophosphate transferases involved in teichoic acid biosynthesis TagF/TagB/Eps (VIMSS-AUTO) | -0.80 | -1.59 |  |
| Dde_3202 393826 Mrp protein (mrp) (VIMSS-AUTO) | -0.80 | -1.47 |  |
| Dde_1711 395065 Flagellar hook protein FlgE | -0.78 | -0.87 |  |
| Dde_2183 394651 dTDP-4-dehydrorhamnose reductase | -0.78 | -0.64 |  |
| Dde_1067 3334169 hypothetical protein | -0.77 | -1.21 |  |
| Dde_3589 393531 Proton conductor component of motor; no effect on switching (VIMSS-AUTO) | -0.75 | -0.83 |  |
| Dde_2384 394475 Response regulator receiver domain protein (VIMSS-AUTO) | -0.73 | -0.56 |  |
| Dde_3200 393828 Iron-sulfur cluster binding protein (VIMSS-AUTO) | -0.70 | -1.34 |  |
| Dde_1031 395643 HD domain, putative (VIMSS-AUTO) | -0.70 | -0.59 |  |
| Dde_0387 393052 Conserved hypothetical protein (VIMSS-AUTO) | -0.68 | -0.92 |  |
| Dde_1459 395296 cytidine deaminase | -0.67 | -1.18 |  |
| Dde_0634 392844 Extracellular solute-binding protein, family 7 (VIMSS-AUTO) | -0.62 | -0.66 |  |
| Dde_3178 393848 Glycerol-3-phosphate dehydrogenase (NAD+) (VIMSS-AUTO) | -0.59 | -1.99 |  |
| Dde_3199 393829 hypothetical protein | -0.51 | -1.06 |  |
|  |  |  |  |
| **Genes shared between 14 Mpa and 3.5 Mpa** | **fitness difference 14 Mpa** | | **fitness difference 3.5 Mpa** |
| Dde_1179 395519 Putative dihydroxyacetone kinase (EC 2.7.1.2) (VIMSS-AUTO) | -0.72 |  | -1.02 |
| Dde_3164 393860 DHH family protein, putative (VIMSS-AUTO) | -0.71 |  | -0.85 |
| Dde_1180 395518 PTS system, fructose-specific EIIA/HPr/EI components (ptsI) (VIMSS-AUTO) | -0.65 |  | -1.46 |
| Dde_1178 395520 Putative dihydroxyacetone kinase (EC 2.7.1.2) (VIMSS-AUTO) | -0.61 |  | -0.74 |
|  |  |  |  |
| **Genes shared between 14, 10.5 and 3.5 Mpa** | **fitness difference 14 Mpa** | **fitness difference 10.5 Mpa** | **fitness difference 3.5 Mpa** |
| Dde_2021 394804 Conserved hypothetical protein | -1.18 | -0.64 | -0.57 |
| Dde_0383 3333988 sigma-70 factor | -1.06 | -0.82 | -0.50 |
| Dde_3158 393866 Flagellar biosynthesis, cell-distal portion of basal-body rod (VIMSS-AUTO) | -0.82 | -1.16 | -0.59 |
| Dde_2673 394235 ferrous iron transporter component feoA (Dmitry Rodionov) | -0.75 | -1.13 | -2.52 |
