# Supplementary material for: Dissimilatory Sulfate Reduction Under High Pressure by Desulfovibrio alaskensis G20
Source: Front Microbiol. 2018 Jul 9;9:1465. doi: 10.3389/fmicb.2018.01465 (PMC6052904; doi:10.3389/fmicb.2018.01465)
Supplement: TABLE S3 — Proteomic data to show proteins up and downregulated in wild-type D. alaskensis G20 at 14 MPa. [file Table_3.DOCX]

**Supporting Information Table 3: Proteomic data to show proteins up- and down-regulated in wild type *D. alaskensis* G20 at 14 MPa**

| **DVU** | **VIMSS no.** | **Description name of protein upregulated at 14 Mpa** | **Proteomics ratio 14 MPa/0.1 Mpa** | |
| --- | --- | --- | --- | --- |
| Dde_0679 |  | Rhodanese-related sulfurtransferase | | 4.4 |
| Dde_1821 |  | Cytochrome C553 precursor | | 3 |
| Dde-1214 |  | Conserved hypothetical protein | | 2.6 |
| Dde_2220 |  | ATP-dependant Clp protease ClpP | | 2.5 |
| Dde_2219 |  | ATP-dependant Clp protease ClpX | |  |
| Dde_1010 |  | Conserved hypothetical protein | | 2.4 |
|  | VIMSS395574 | QmoC Quinone-interacting membrane-bound oxidoreductase | | 2.15 |
|  | VIMSS394393 | mopB GroES, 10 Kd chaperone binds to Hsp60 in pres. Mg-ATP, suppressing its ATPase activity | | 2.12 |
| Dde_1442 | VIMSS395310 | Protease IV, putative | | 1.94 |
| Dde_0152 | VIMSS3333939 | DEAD/DEAH box helicase domain protein (NCBI) | | 1.92 |
| Dde_1689 |  | OmpA family domain protein | | 1.89 |
|  | VIMSS394593 | rpsQ 30S ribosomal subunit protein S17 | | 1.88 |
| Dde_2169 | VIMSS394665 | UptF, putative | | 1.88 |
|  | VIMSS394850 | aspC4 Aminotransferase, class I (aspC4) | | 1.84 |
| Dde_3023 | VIMSS392931 | dsrD Dissimilatory sulfite reductase D (Shelley Haveman) | | 1.81 |
| Dde_0589 | VIMSS392875 | Putative regulator | | 1.80 |
|  | VIMSS393488 | pal Peptidoglycan-associated lipoprotein | | 1.71 |
|  | VIMSS394668 | ilvC Ketol-acid reductoisomerase / Dehydropantoate reductase (Natalia Ivanova) | | 1.70 |
| Dde_3413 | VIMSS393668 | ATPases with chaperone activity, ATP-binding subunit | | 1.69 |
| Dde_3195 | VIMSS393832 | fprA-2 Putative flavodoxin | | 1.68 |
|  | VIMSS394013 | tuf Translation elongation factor Tu | | 1.66 |
|  | VIMSS394007 | rplJ 50S ribosomal subunit protein L10 | | 1.65 |
| Dde_3736 | VIMSS393401 | gap Glyceraldehyde-3-phosphate dehydrogenase, type I | | 1.64 |
| Dde_0972 | VIMSS395695 | ftsA-3 Member of the coenzyme F390 synthetase / phenylacetate CoA ligase family | | 1.62 |
| Dde_3412 | VIMSS393669 | Molecular chaperone (small heat shock protein) | | 1.55 |
| Dde_1312 | VIMSS395426 | Copper chaperone | | 1.50 |
|  | VIMSS395339 | rplI Ribosomal protein L9 | | 1.47 |
|  | VIMSS394995 | glpX Fructose-1,6-bisphosphatase, class II | | 1.46 |
|  | VIMSS393791 | pta Phosphotransacetylase | | 1.39 |
|  | VIMSS393793 | glcD Glycolate oxidase subunit D | | 1.39 |
|  | VIMSS392948 | rpsA Ribosomal protein S1 | | 1.38 |
|  |  |  | |  |
| **DVU** | **VIMSS no.** | **Description name of protein downregulated at 14 Mpa** | **Proteomics ratio 14 MPa/0.1 Mpa** | |
| Dde_2335 | VIMSS394519 | Conserved hypothetical protein | | 0.85 |
| Dde_1407 | VIMSS395342 | Putative protease maturation protein | | 0.69 |
|  | VIMSS394745 | pyc Pyruvate carboxylase (pyc) | | 0.62 |
| Dde_3707 | VIMSS3334833 | hypothetical protein (NCBI) | | 0.60 |
|  | VIMSS395092 | thiC thiamine biosynthesis protein ThiC (RefSeq) | | 0.56 |
| Dde_2857 | VIMSS394094 | Chemoreceptor protein a | | 0.42 |
| Dde_3103 | VIMSS3334667 | Sirohydrochlorin cobaltochelatase (NCBI) | | 0.40 |
| Dde_1281 | VIMSS395446 | Putative ATP-binding component of a transport system | | 0.40 |
|  | VIMSS394184 | hom Homoserine dehydrogenase (hom) | | 0.35 |
|  | VIMSS395683 | atpG ATP synthase F1, gamma subunit | | 0.32 |
|  | VIMSS393135 | eno enolase (RefSeq) | | 0.28 |
| Dde_2702 | VIMSS394212 | DNA-binding protein | | 0.24 |
| Dde_1444 | VIMSS395308 | ABC transporter subunit | | 0.24 |
|  | VIMSS394263 | infC Translation initiation factor IF-3 (NCBI) | | 0.22 |
| Dde_0520 | VIMSS392937 | 2-phosphinomethylmalic acid synthase, putative | | 0.22 |
| Dde_1253 | VIMSS395469 | Malic enzyme | | 0.22 |
| Dde_1207 | VIMSS395494 | Heterodisulfide reductase subunit C | | 0.20 |
|  | VIMSS393367 | gyrA DNA gyrase, A subunit | | 0.20 |
| Dde_3635 | VIMSS393487 | gltA Glutamate synthase (NADPH), homotetrameric | | 0.20 |
| Dde_0104 | VIMSS393294 | glnA Glutamine synthetase, type I | | 0.17 |
|  | VIMSS394285 | gatB Glutamyl-tRNA(Gln) amidotransferase, B subunit | | 0.17 |
| Dde_2317 | VIMSS394533 | Acetyl-CoA synthetase, putative | | 0.17 |
| Dde_2037 | VIMSS394788 | Peptidyl-prolyl cis-trans isomerase family protein, putative | | 0.17 |
|  | VIMSS395267 | cbhK PfkB family carbohydrate kinase family (cbhK) | | 0.17 |
|  | VIMSS394792 | glyS Glycine tRNA synthetase, beta subunit | | 0.15 |
| Dde_2075 | VIMSS394751 | ATP-binding protein | | 0.15 |
|  | VIMSS395560 | frr ribosome recycling factor (RefSeq) | | 0.14 |
| Dde_0248 | VIMSS393179 | dnaJ Chaperone with DnaK; heat shock protein | | 0.13 |
| Dde_2129 | VIMSS394699 | CarD-like transcriptional regulator superfamily | | 0.13 |
| Dde_1368 | VIMSS395378 | Orf, hypothetical protein | | 0.11 |
|  | VIMSS394267 | pheT Phenylalanyl-tRNA synthetase, beta subunit | | 0.10 |
|  | VIMSS393222 | serS seryl-tRNA synthetase (RefSeq) | | 0.09 |
| Dde_3201 | VIMSS393827 | Iron-sulfur cluster binding protein | | 0.07 |
| Dde_1255 | VIMSS395467 | Fumarate hydratase, class I, putative | | 0.06 |
| Dde_1256 | VIMSS395466 | frdB fumarate reductase, iron sulfur protein (Jessica Butler) | | 0.05 |
| Dde_1257 | VIMSS395465 | frdA Fumarate reductase, anaerobic, flavoprotein subunit | | 0.04 |
