# Supplementary material for: Dissimilatory Sulfate Reduction Under High Pressure by Desulfovibrio alaskensis G20
Source: Front Microbiol. 2018 Jul 9;9:1465. doi: 10.3389/fmicb.2018.01465 (PMC6052904; doi:10.3389/fmicb.2018.01465)
Supplement: TABLE S4 — Venn analysis of data from competitive fitness experiments to show beneficial mutations shared between all pressures tested. [file Table_4.DOCX]

**Supporting Information Table 4: Venn analysis of data from competitive fitness experiments to show beneficial mutations shared between all pressures tested**

| **Genes shared between 14 Mpa and 3.5 Mpa** | **fitness difference 14 Mpa** | | **fitness difference 3.5 Mpa** |
| --- | --- | --- | --- |
| Dde_2168 394666 Acetolactate synthase, large subunit, biosynthetic type (VIMSS-AUTO) | 1.77 |  | 4.81 |
| Dde_2049 394776 2-isopropylmalate synthase/homocitrate synthase family protein (VIMSS-AUTO) | 1.46 |  | 5.15 |
| Dde_0538 392920 Conserved hypothetical protein (VIMSS-AUTO) | 1.39 |  | 3.19 |
| Dde_3016 393986 Anaerobic ribonucleoside-triphosphate reductase (VIMSS-AUTO) | 1.18 |  | 1.08 |
| Dde_2366 394491 Flp pilus assembly protein TadD, contains TPR repeats (VIMSS-AUTO) | 1.11 |  | 0.53 |
| Dde_0334 393101 amidophosphoribosyltransferase | 1.00 |  | 4.56 |
| Dde_0314 393119 Conserved hypothetical protein TIGR00250 (VIMSS-AUTO) | 0.99 |  | 2.58 |
| Dde_1209 395492 Heterodisulfide reductase subunit (VIMSS-AUTO) | 0.99 |  | 0.64 |
| Dde_1564 395197 Helicase, Snf2 family (VIMSS-AUTO) | 0.96 |  | 2.29 |
| Dde_3678 393453 Glycosyltransferases, probably involved in cell wall biogenesis (VIMSS-AUTO) | 0.94 |  | 2.98 |
| Dde_3596 393525 Aspartate aminotransferase (aspB) (VIMSS-AUTO) | 0.78 |  | 3.03 |
| Dde_2323 394528 Holliday junction DNA helicase RuvA (VIMSS-AUTO) | 0.78 |  | 5.94 |
| Dde_0040 393342 Conserved hypothetical protein (VIMSS-AUTO) | 0.76 |  | 1.47 |
| Dde_0572 392889 Carboxynorspermidine synthase (Natalia Ivanova) | 0.73 |  | 1.47 |
| Dde_0254 393173 Aspartate-semialdehyde dehydrogenase (VIMSS-AUTO) | 0.73 |  | 1.26 |
| Dde_0540 392918 Coenzyme F390 synthetase family (COG1541) (Morgan Price) | 0.73 |  | 2.74 |
| Dde_3134 393884 Adenosylhomocysteinase (VIMSS-AUTO) | 0.71 |  | 0.79 |
| Dde_2324 394527 Crossover junction endodeoxyribonuclease RuvC (VIMSS-AUTO) | 0.71 |  | 5.98 |
| Dde_1207 395494 Heterodisulfide reductase subunit C (VIMSS-AUTO) | 0.69 |  | 1.99 |
| Dde_1213 395488 2-polyprenylphenol hydroxylase and related flavodoxin oxidoreductases (VIMSS-AUTO) | 0.68 |  | 1.48 |
| Dde_1208 395493 Succinate dehydrogenase, subunit C (VIMSS-AUTO) | 0.66 |  | 0.78 |
| Dde_0289 393140 Transcriptional regulator containing PAS, AAA-type ATPase, and DNA-binding domains (VIMSS-AUTO) | 0.65 |  | 0.71 |
| Dde_3635 393487 Glutamate synthase (NADPH), homotetrameric (VIMSS-AUTO) | 0.65 |  | 0.61 |
| Dde_3774 393380 Conserved hypothetical protein (VIMSS-AUTO) | 0.64 |  | 6.40 |
| Dde_1211 395490 hydrogenase, iron-sulfur cluster-binding subunit, putative | 0.60 |  | 1.33 |
| Dde_3681 393450 Sialic acid synthase (VIMSS-AUTO) | 0.59 |  | 1.98 |
| Dde_3104 393911 Citrate-dependent iron transport, membrane-bound protein (VIMSS-AUTO) | 0.58 |  | 2.37 |
| Dde_2220 394616 ATP-dependent Clp protease, proteolytic subunit ClpP (VIMSS-AUTO) | 0.56 |  | 2.83 |
| Dde_3677 393454 Asparagine synthase (glutamine-hydrolyzing) (VIMSS-AUTO) | 0.55 |  | 3.62 |
| Dde_2373 394484 RecA protein (VIMSS-AUTO) | 0.54 |  | 6.77 |
| Dde_3773 393381 hypothetical protein | 0.53 |  | 5.01 |
| Dde_1773 395017 Phosphotransferase system enzyme IIA, regulates N metabolism (VIMSS-AUTO) | 0.52 |  | 0.64 |
| Dde_2704 394210 Cobalamin 5'-phosphate synthase (VIMSS-AUTO) | 0.51 |  | 1.54 |
|  |  |  |  |
| **Genes shared between 10.5 Mpa and 3.5 Mpa** |  | **fitness difference 10.5 Mpa** | **fitness difference 3.5 Mpa** |
| Dde_3679 393452 Spore coat polysaccharide biosynthesis protein F, CMP-KDO synthetase homolog (VIMSS-AUTO) |  | 0.76 | 1.49 |
| Dde_1255 395467 Fumarate hydratase, class I, putative (VIMSS-AUTO) |  | 0.69 | 0.68 |
| Dde_0521 392936 Conserved hypothetical protein |  | 0.68 | 1.31 |
| Dde_2092 394735 dihydrodipicolinate reductase |  | 0.66 | 3.39 |
| Dde_2144 3334415 hypothetical protein |  | 0.64 | 1.61 |
| Dde_1260 395463 Sigma-54 dependent DNA-binding response regulator (VIMSS-AUTO) |  | 0.63 | 0.52 |
| Dde_1808 394983 Conserved hypothetical protein (VIMSS-AUTO) |  | 0.60 | 0.52 |
| Dde_0014 393361 methionyl-tRNA formyltransferase |  | 0.59 | 1.57 |
| Dde_2306 394543 Arginine ABC transporter, periplasmic arginine-binding protein, putative (VIMSS-AUTO) |  | 0.58 | 0.59 |
| Dde_1195 395504 Nitroreductase (VIMSS-AUTO) |  | 0.56 | 1.09 |
| Dde_2945 394052 Phosphomannomutase / phosphoglucomutase (Natalia Ivanova) |  | 0.53 | 4.92 |
| Dde_3676 393455 Acetyltransferase (isoleucine patch superfamily) (VIMSS-AUTO) |  | 0.52 | 1.34 |
| Dde_2296 394552 Conserved hypothetical protein (VIMSS-AUTO) |  | 0.52 | 1.37 |
|  |  |  |  |
| **Genes shared between 14, 10.5 and 3.5 Mpa** | **fitness difference 14 Mpa** | **fitness difference 10.5 Mpa** | **fitness difference 3.5 Mpa** |
| Dde_2113 394715 Acetyltransferase, GNAT family, putative (VIMSS-AUTO) | 1.02 | 0.55 | 1.88 |
| Dde_2145 394685 Periplasmic protein, putative (VIMSS-AUTO) | 0.92 | 0.68 | 1.95 |
| Dde_0358 393079 NAD-dependent epimerase/dehydratase family protein | 0.83 | 0.54 | 2.32 |
| Dde_3675 393456 hypothetical protein | 0.67 | 0.63 | 3.33 |
| Dde_0937 395724 hypothetical protein | 0.62 | 0.53 | 1.23 |
| Dde_1688 395085 Conserved hypothetical protein (VIMSS-AUTO) | 0.62 | 0.65 | 4.50 |
