# Supplementary material for: Dissimilatory Sulfate Reduction Under High Pressure by Desulfovibrio alaskensis G20
Source: Front Microbiol. 2018 Jul 9;9:1465. doi: 10.3389/fmicb.2018.01465 (PMC6052904; doi:10.3389/fmicb.2018.01465)
Supplement: TABLE S5 — Average growth rate and maximum OD600 of Desulfovibrio alaskensis G20 wild type (wt), and transposon mutants flaB3, fliD and fliA at 0.1 MPa and 14 MPa. [file Table_5.docx]

**Supporting Information Table 5: Average growth rate and maximum OD_600_ of *Desulfovibrio alaskensis* G20 wild type (*wt*), and transposon mutants *flaB3, fliD and fliA* at 0.1 MPa and 14 MPa.**

| **Strain/**  **mutant** | **Average growth rate 0.1 MPa hr^-1^** | **Average growth rate 14 MPa hr^-1^** | **Average max OD 0.1 Mpa** | **Average max OD 14 Mpa** |
| --- | --- | --- | --- | --- |
| ***wt*** | 0.062 ± 0.003 | 0.046 ± 0.007 | 0.72 ± 0.03 | 0.70 ± 0.04 |
| ***flaB3*** | 0.051 ± 0.007 | 0.018 ± 0.007 | 0.71 ± 0.01 | 0.54 ± 0.03 |
| ***fliD*** | 0.051 ± 0.004 | 0.025 ± 0.003 | 0.71 ± 0.01 | 0.53 ± 0.01 |
| ***fliA*** | 0.052 ± 0.004 | 0.023 ± 0.002 | 0.73 ± 0.02 | 0.54 ± 0.01 |
